# Pronecroptotic Therapy Using Ceramide Nanoliposomes Is Effective for Triple-Negative Breast Cancer Cells

**DOI:** 10.3390/cells13050405

**Published:** 2024-02-26

**Authors:** Yuki Ohya, Yuri Ogiso, Masaya Matsuda, Harumi Sakae, Kentaro Nishida, Yasuhiro Miki, Todd E. Fox, Mark Kester, Wataru Sakamoto, Takeshi Nabe, Kazuyuki Kitatani

**Affiliations:** 1 Faculty of Pharmaceutical Sciences, Setsunan University, Hirakata 573-0101, Japan23d402oy@edu.setsunan.ac.jp (Y.O.); masaya.matsuda@pharm.setsunan.ac.jp (M.M.); 21d401sh@edu.setsunan.ac.jp (H.S.); kentaro.nishida@pharm.setsunan.ac.jp (K.N.); t-nabe@pharm.setsunan.ac.jp (T.N.); 2 Department of Pathology, Tohoku University Graduate School of Medicine, Sendai 980-8575, Japan; 3 Department of Pharmacology, University of Virginia, Charlottesville, VA 22908-8735, USA; 4 Research Center of Oncology, Ono Pharmaceutical, Co., Ltd., Osaka 618-8585, Japan; w.sakamoto@ono-pharma.com

**Keywords:** breast cancer, ceramide, ceramide nanoliposome, mixed-lineage kinase domain-like protein, necroptosis, triple-negative breast cancer

## Abstract

Regulated necrosis, termed necroptosis, represents a potential therapeutic target for refractory cancer. Ceramide nanoliposomes (CNLs), considered potential chemotherapeutic agents, induce necroptosis by targeting the activating protein mixed lineage kinase domain-like protein (MLKL). In the present study, we examined the potential of pronecroptotic therapy using CNLs for refractory triple-negative breast cancer (TNBC), for which there is a lack of definite and effective therapeutic targets among the various immunohistological subtypes of breast cancer. MLKL mRNA expression in tumor tissues was significantly higher in TNBC patients than in those with non-TNBC subtypes. Similarly, among the 50 breast cancer cell lines examined, MLKL expression was higher in TNBC-classified cell lines. TNBC cell lines were more susceptible to the therapeutic effects of CNLs than the non-TNBC subtypes of breast cancer cell lines. In TNBC-classified MDA-MB-231 cells, the knockdown of MLKL suppressed cell death induced by CNLs or the active substance short-chain C_6_-ceramide. Accordingly, TNBC cells were prone to CNL-evoked necroptotic cell death. These results will contribute to the development of CNL-based pronecroptotic therapy for TNBC.

## 1. Introduction

Breast cancer is the most commonly diagnosed type of cancer in women and the second highest cause of cancer-related death. In GLOBOCAN 2020, the incidence and mortality of breast cancer were reported to be 2,261,419 new cases and 684,996 deaths, respectively [1]. Breast cancer is a genetically and clinically heterogeneous disease that is clinically classified into four subtypes by the expression of receptors, such as the estrogen receptor (ER), progesterone receptor (PR), and human epidermal growth factor receptor 2 (HER2). Subclassification is fundamental for personalized pharmacotherapy [2,3]. Among the subtypes, triple-negative breast cancer (TNBC), which is defined by the lack of ER and PR expression and the absence of HER2 amplification, accounts for 10–20% of annually diagnosed breast cancer cases [4]. The clinical features of TNBC patients include high invasiveness, a high metastatic potential, proneness to relapse, and poor prognoses [4]. The primary therapeutic regimen for TNBC is limited to radiotherapy and conventional chemotherapy because of the lack of therapeutic molecular targets, such as ER, PR, and HER2 [2,4]. Therefore, novel therapeutic targets need to be developed. 

Necroptosis is a form of regulated cell death with the hallmarks of necrosis and is a therapeutic target for cancer treatment [5,6,7]. The induction of necroptosis has attracted attention as an alternative approach for cancer treatment to overcome chemoresistance and promote anti-tumor immunity [8,9,10]. The signaling pathway of necroptosis includes regulatory proteins, such as receptor-interacting protein kinase (RIPK) 1 and RIPK3, and activating pore-forming mixed lineage kinase domain protein (MLKL). RIPK3 phosphorylates MLKL, and phosphorylated MLKL, in turn, forms an oligomer that translocates to the plasma membrane to form pores, which eventually increase plasma membrane permeability [7,11,12]. Epigenetically, RIPK3 expression is often silenced in cancer cells [13]. MLKL expression is transcriptionally up-regulated by interferon-γ-regulatory factor 1 (IRF1) and the signal transducer and activator of transcription 1 (STAT1) in MDA-MB-231 breast cancer and HeLa cervical cancer cell lines [14]. The up-regulation of MLKL is presumed to sensitize cancer cells to necroptotic cell death, and pronecroptotic therapy is potentially effective for cancer cells that highly express MLKL.

Ceramide is a central lipid in sphingolipid metabolism and a potent tumor suppressor that augments apoptotic and non-apoptotic cell death [15,16,17,18]. The potential of ceramide-based therapeutics for the treatment of cancer has been attracting increasing interest [19,20,21,22]. We developed a non-toxic and biologically stable nanoliposome formulation of short-chain C_6_-ceramide termed ceramide nanoliposomes (CNLs) as potential chemotherapeutic agents [22]. CNLs exerted therapeutic effects in preclinical cancer models [23,24,25] and are currently being investigated clinically [26]. 

CNLs activate MLKL-governed necroptosis independent of RIPK3 in ovarian cancer cells [25]. Importantly, the anti-tumor activities of CNLs correlated with MLKL expression in ovarian cancer cell lines, implying the involvement of MLKL expression in the therapeutic effectiveness of CNLs. In the present study, we investigated the potential of pronecroptotic therapy for breast cancer using CNLs.

## 2. Materials and Methods

### 2.1. Materials 

Horseradish peroxidase-conjugated antibodies against mouse or rabbit IgG and human serum were purchased from Jackson ImmunoResearch (West Grove, PA, USA). Mouse monoclonal antibodies specific for β-actin (clone 15G5A11/E2, MA1-140), SuperSignal West Dura Extended Duration Substrate, Triton X-100, BCA protein assay reagents, OPTI MEM, goat anti-rabbit IgG conjugated with Alexa555, RNAiMAX, control siRNAs (4390843 or 4390846), and human MLKL siRNAs (MLKL siRNA-1, s47088; MLKL siRNA-2, s47089) were obtained from Thermo Fisher Scientific (Rockford, IL, USA). Roswell Park Memorial Institute 1640 medium, high glucose Dulbecco’s Modified Eagle’s Medium, phosphate-buffered saline (PBS), EDTA, glycine, NaCl, Tris, sodium dodecyl sulfate (SDS), and trypsin were purchased from Nacalai Tesque (Kyoto, Japan). Fetal bovine serum was obtained from Biowest (Nuaillé, France). A rabbit anti-MLKL antibody (ab184718) was purchased from Abcam (Cambridge, MA, USA). Gradient polyacrylamide gels (4–20%) and nitrocellulose membranes were obtained from Bio-Rad (Hercules, CA, USA). 4’,6-Diamidino-2-phenylindole was obtained from Dojindo (Kumamoto, Japan). C_6_-Ceramide (*N*-hexanoyl-*D*-erythro-sphingosine) was purchased from Avanti Polar Lipids (Alabaster, AL, USA). Glass bottom culture dishes (35 mm) were from MATTEK (Ashland, MA, USA). MDA-MB-231, MDA-MB-453, MDA-MB-468, MCF-7, ZR-751, T47D, HCC1937, and BT-20 cells were obtained from ATCC (Manassas, VA, USA). CellTiter-Glo2.0 was purchased from Promega (Madison, WI, USA).

### 2.2. Mixed Lineage Kinase Domain-like Protein Expression Profiles of Breast Cancer Tissues

To examine MLKL expression in the tumor tissues of patients with breast cancer, we searched The Cancer Genome Atlas (TCGA) database obtained from cBioportal (https://www.cbioportal.org/) and characterized immunohistological subtypes.

### 2.3. Gene Expression Profiles of Breast Cancer Cell Lines

Breast cancer cell lines were subclassified into two groups: TNBC and non-TNBC. Twenty-four TNBC cell lines (BT20, BT-549, CAL120, CAL148, CAL51, CAL851, DU4475, HCC1143, HCC1187, HCC1395, HCC1599, HCC1806, HCC1937, HCC2157, HCC38, HCC70, HDQ-P1, HMC18, HS578T, MDA-MB-157, MDA-MB-231, MDA-MB-436, MDA-MB-453, and MDA-MB-468) were clinicopathologically characterized and subclassified [27,28,29]. The remaining non-TNBC cell lines (AU565, BT-474, BT-483, CAMA1, EFM19, EFM192A, HCC1419, HCC1428, HCC1500, HCC1569, HCC1954, HCC202, HCC2218, KPL1, MCF-7, MDA-MB-134VI, MDA-MB-175VII, MDA-MB-361, MDA-MB-415, SKBR3, T47D, JIMT1, UACC812, UACC893, ZR751, and ZR7530) were subclassified in accordance with the DepMap (https://depmap.org/portal/)-based gene expression profiles of ER, PR, and HER2.

### 2.4. Cell Culture

MDA-MB-231, MDA-MB-453, and MDA-MB-468 cells were cultured in Dulbecco’s Modified Eagle’s Medium supplemented with 10% fetal bovine serum. MCF-7, ZR-751, T47D, HCC1937, and BT-20 cells were cultured in Roswell Park Memorial Institute 1640 medium supplemented with 10% fetal bovine serum. Cells were maintained at <80% confluence under standard incubator conditions (37 °C in a humidified atmosphere with 5% CO_2_).

### 2.5. Ceramide Nanoliposomes Preparation

CNLs were prepared as described in Sakae et al. [30]. Briefly, dioleoylphosphatidylethanolamine, distearoylphosphatidylcholine, distearoylphosphatidylethanolamine-polyethylene glycol 2000, polyethylene glycol 750-N-octanoylsphingosine, and C_6_-ceramide were mixed in chloroform, dried to a thin film under nitrogen, and then hydrated by the addition of sterilized 0.9% NaCl solution at 60 °C with sonicating and vortexing. Lipid solutions were then extruded at 60 °C by passing through 100 nm polycarbonate filters. Size (80 nM) and charge (−7 mV) were validated using Malvern Zetasizer Nano (Malvern Panalytical, Malvern, UK). 

### 2.6. Cell Viability Assay

Cells (4 × 10^3^ cells/well) seeded on a 96-well culture plate were treated with ceramide-free ghost nanoliposomes or CNLs for 24 h. Cell viability was assessed using the CellTiter-Glo luminescent cell viability assay in accordance with the manufacturer’s protocol. Luminescence was measured with a GloMax Navigator Microplate Luminometer (Promega, Madison, WI, USA).

### 2.7. siRNA Transfection

Cells were seeded in 6-well plates (4 × 10^5^ cells/well) and transfected with 5 nM siRNAs for control or MLKL using RNAiMAX transfection reagent in accordance with the manufacturer’s instructions. For each well, 5 pmol of siRNAs, 150 μL of OPTI MEM, and 1 μL of RNAiMAX were used. After transfection for 24 h, cells were treated with CNL or ghost nanoliposomes. 

### 2.8. Trypan Blue Exclusion Assay

Harvested cells were resuspended in 100 µL of PBS. A 1:1 dilution of a cell suspension (15 μL) and 0.4% trypan blue solution (15 μL) was prepared and loaded into a hemocytometer. The numbers of trypan blue-excluding live cells and dead cells were counted. 

### 2.9. Immunofluorescence Microscopy

MDA-MB-231 cells (1 or 2 × 10^5^ cells) were seeded on 35 mm glass-bottomed dishes. Cells were fixed with 4% formaldehyde at room temperature for 10 min. Fixed cells were further permeabilized with 0.1% Triton X-100 for 10 min. After washing with PBS, cells were blocked at room temperature for 1 h with 20% human serum. Cells were incubated with an MLKL antibody (1:500 dilution) in PBS containing 20% human serum overnight at 4 °C. After washing with PBS, cells were incubated with an Alexa555-conjugated anti-rabbit IgG antibody (1:500 dilution) at 4 °C for 1 h. Nuclear staining was performed with 4’,6-diamidino-2-phenylindole (1:1000 dilution). All images were captured using a confocal microscope (Olympus FV1000D system, Olympus, Tokyo, Japan).

### 2.10. Western Blotting

Cells were harvested with ice-cold PBS containing 10 mM EDTA. The protein contents of samples were measured by a BCA protein assay. Proteins were denatured at 98 °C for 10 min. Proteins (5 μg) were subjected to SDS-polyacrylamide gel electrophoresis (4–20% gradient polyacrylamide gels) and then electrophoretically transferred onto nitrocellulose membranes. Membranes were blocked with PBS/0.1% Tween 20 containing 5% nonfat dried milk at room temperature for 30 min. After washing with PBS/0.1% Tween 20, membranes were incubated with antibodies against MLKL (1:1000) or β-actin (1:10,000) in PBS/0.1% Tween 20 containing 5% bovine serum albumin at 4 °C overnight. Membranes were washed with PBS/0.1% Tween 20 and incubated with secondary antibodies conjugated with horseradish peroxidase in PBS/0.1% Tween 20 containing 5% nonfat dried milk at 4 °C for 1 h. After washing, proteins were visualized using enhanced chemiluminescence reagents. The quantification of chemiluminescent signals was performed with a ChemiDoc digital imaging system (Bio-Rad, Hercules, CA, USA).

### 2.11. Statistical Analysis

Data were analyzed using multiple *t*-tests and a robust or simple linear regression by GraphPad Prism software version 8.4.3. *p* values < 0.05 were considered to be significant.

## 3. Results

### 3.1. High Mixed Lineage Kinase Domain-like Protein Expression in Triple-Negative Breast Cancer Patients

**Figure 1:**
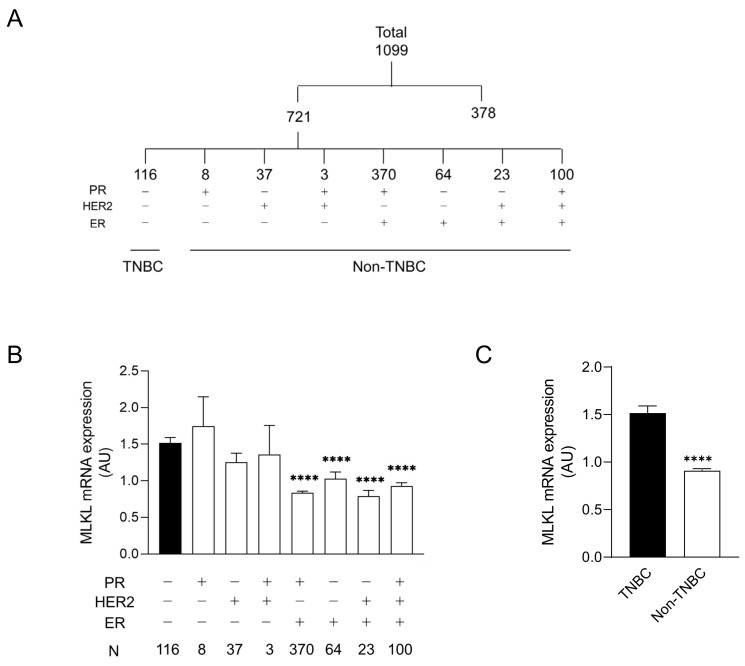
mRNA expression of mixed lineage kinase domain-like protein (MLKL) in tumor tissues of patients with breast cancer. (**A**) Among 1099 patients registered in The Cancer Genome Atlas (TCGA), 721 were divided into eight molecular subtypes by the expression of receptors, including estrogen receptor (ER), progesterone receptor (PR), and human epidermal growth factor receptor 2 (HER2). (**B**) Eight subgroups were further divided into two groups: triple-negative breast cancer (TNBC) and the remaining seven molecular subtypes (non-TNBC). Data represent means ± standard error. ****, *p* < 0.0001, compared to TNBC. (**C**) MLKL mRNA expression in tumor tissues was compared between TNBC and non-TNBC. Data represent means ± standard error. ****, *p* < 0.0001.

CNLs are regarded as pronecroptotic chemotherapeutic reagents, and MLKL is predictive of the therapeutic effectiveness of CNLs. To predict the susceptibility of the immunohistological subtypes of breast cancer to CNL-induced necroptosis, MLKL expression in tumor tissues was analyzed using the TCGA database. Among 1099 cases, 721 were defined as “positive” and/or “negative” for ER, PR, and HER2 based on immunohistological profiles in the TCGA database. The profiles of the remaining 378 cases were not immunohistologically subclassified because of (1) full or partial defects in the profile in the TCGA database or (2) they were defined as “equivocal” and/or “indeterminate”. The 721 cases were further divided into eight subtypes by distinct receptor expression (Figure 1A). Figure 1B shows MLKL mRNA expression in distinct subtypes. A statistical analysis of seven subtypes relative to TNBC was performed. MLKL mRNA expression was significantly higher in the TNBC group than in the PR (+)/HER2 (−)/ER (+), PR (−)/HER2 (−)/ER (+), PR (−)/HER2 (+)/ER (+), and PR (+)/HER2 (+)/ER (+) groups. MLKL mRNA expression was also significantly higher in the TNBC group than in the non-TNBC groups (Figure 1C). 

To obtain an understanding of the relevance of MLKL mRNA expression to the prognosis of patients with breast cancer, we performed an online analysis with Kaplan–Meier Plotter (http://www.kmplot.com) [31]. Patients were divided into two groups: high and low MLKL expression. Progression-free survival periods were plotted to generate Kaplan–Meier curves. As shown in Supplementary Figure S1, progression-free survival was longer in the MLKL-high patient group than in the MLKL-low group. MLKL expression in tumor tissues can possibly affect therapeutic effectiveness.

### 3.2. High Mixed Lineage Kinase Domain-like Protein Expression in Triple-Negative Breast Cancer Cell Lines

**Figure 2:**
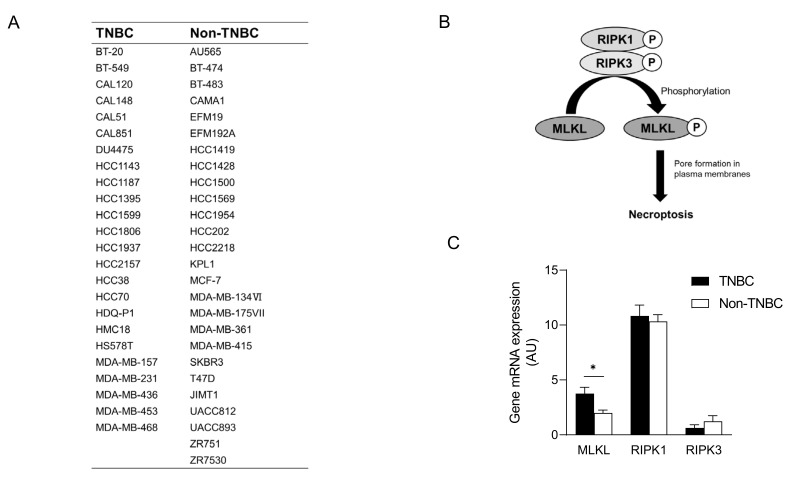
mRNA expression of mixed lineage kinase domain-like protein (MLKL) in breast cancer cell lines. (**A**) List of breast cancer cell lines and their subclassification into triple-negative breast cancer (TNBC) (*n* = 24) and non-TNBC (*n* = 26). (**B**) Molecular mechanisms underlying necroptosis. (**C**) MLKL mRNA expression was compared between TNBC and non-TNBC. Data represent means ± standard error. *, *p* < 0.05 by the Student’s *t*-test.

**Figure 3:**
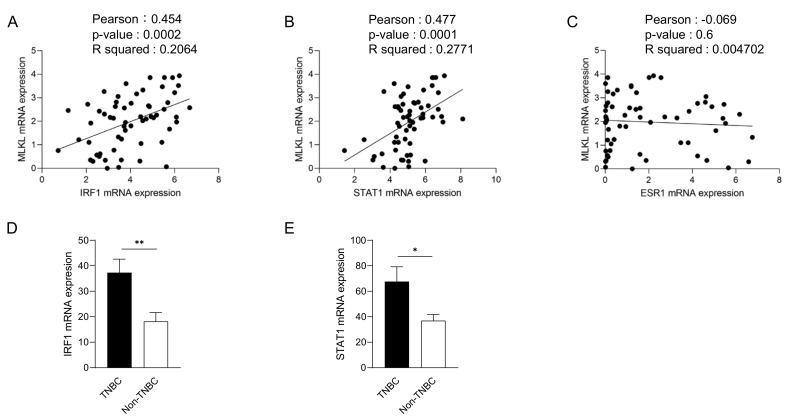
Correlations between mixed lineage kinase domain-like protein (MLKL) mRNA expression and estrogen receptor 1 (ESR1), interferon regulatory factor 1 (IRF1) or signal transducer and activator of transcription 1 (STAT1) mRNA expression in breast cancer cell lines. The mRNA expression of ESR1, IRF1, or STAT1 is plotted with MLKL mRNA expression. *R^2^* values were obtained using GraphPad Prism (**A**–**C**). (**D**,**E**) IRF1 (**D**) and STAT1 (**E**) mRNA expression was compared between TNBC and non-TNBC. Data represent means ± standard error. *, *p* < 0.05; **, *p* < 0.01.

To recapitulate the high expression of MLKL in TNBC, we investigated MLKL mRNA expression in breast cancer cell lines. These cell lines were subclassified into TNBC (*n* = 24) [27,28,29] and non-TNBC (*n* = 26), and the gene expression profiles of the cell lines were obtained from DepMap (Figure 2A). In addition to MLKL, the necroptosis-associated genes RIPK1 and RIPK3 (Figure 2B) were also examined. MLKL gene expression was significantly higher in TNBC than in the non-TNBC; however, no significant differences were observed in RIPK1 and RIPK3 expression (Figure 2C). To investigate the possible mechanisms by which MLKL is up-regulated in TNBC cells, further database searches were performed. The STAT1-IRF1 pathway transcriptionally promotes MLKL expression in multiple types of cancer cell lines in response to interferon-γ [14]. MLKL mRNA expression correlated with IRF1 and STAT1 mRNA expression, but not with ESR1 expression, in breast cancer cell lines (Figure 3A–C). Moreover, the mRNA expression of IRF1 and STAT1 was significantly higher in TNBC than in the non-TNBC (Figure 3D,E). Therefore, the STAT1-IRF1 pathway was assumed to contribute to the high expression of MLKL in TNBC cells.

### 3.3. Therapeutic Effects of Ceramide Nanoliposomes in Breast Cancer Cell Lines

**Figure 4:**
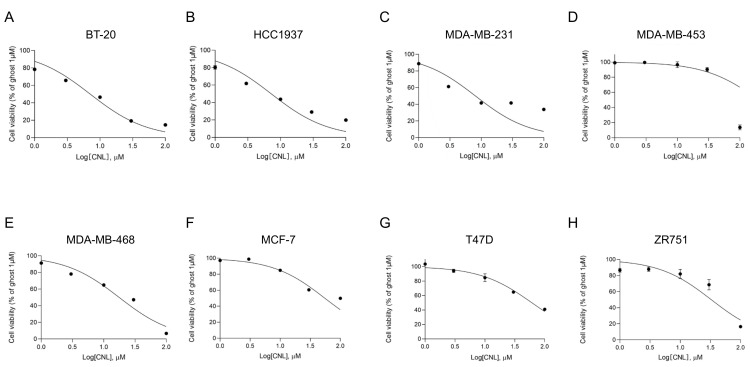
Effects of ceramide nanoliposomes (CNLs) on the cell viability of breast cancer cell lines. BT-20 (**A**), HCC1937 (**B**), MDA-MB-231 (**C**), MDA-MB-453 (**D**), MDA-MB-468 (**E**), MCF-7 (**F**), T47D (**G**), or ZR751 (**H**) cells were seeded on 96-well plates (4000 cells/well). Cells were treated with CNLs up to 100 µM or 1 µM ceramide-free ghost nanoliposomes for 24 h. Cell viability was assessed using a CellTiter-Glo luminescent cell viability assay. Cell viability is expressed as the percentage of 1 μM ceramide-free ghost nanoliposomes. Data represent means ± standard error (*n* = 3).

In ovarian cancers, MLKL increases the susceptibility of cells to necroptosis induced by CNLs [25]. To assess the susceptibility of MLKL-high TNBC cell lines to CNLs, we examined the effects of CNLs on the viability of breast cancer cell lines (Figure 4) and measured IC_50_ values (Table 1). Among the TNBC cell lines examined, the IC_50_ values of BT-20, HCC1937, MDA-MB-231, and MDA-MB-468 cells were < 20 μM, whereas that of MDA-MB-453 cells was > 200 μM. Notably, TNBC cell lines, except for MDA-MB-453 cells, showed lower IC_50_ values than the non-TNBC subtypes, including MCF-7, T47D, and ZR751 cells. The susceptibility of TNBC cell lines to CNL-induced cell death appeared to be higher than that of the non-TNBC cell lines.

### 3.4. Pronecroptotic Effects of Ceramide Nanoliposomes in Triple-Negative Breast Cancer Cell Lines

**Figure 5:**
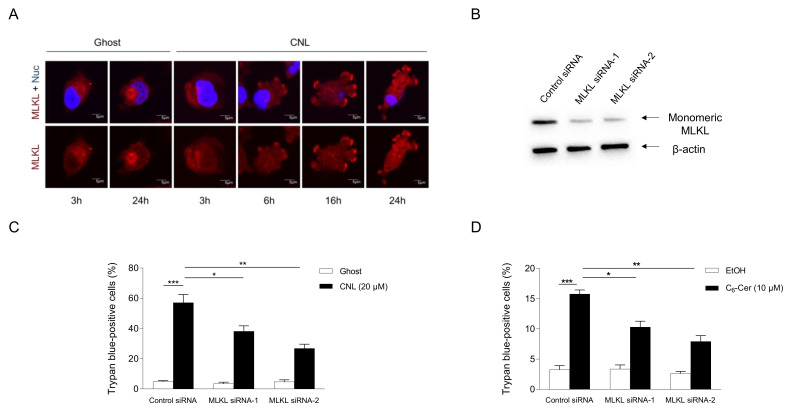
Ceramide involvement in necroptosis. MDA-MB-231 cells (1 × 10^5^ cells/35 mm glass-bottomed dish) were treated with 20 μM ceramide nanoliposomes (CNLs) or ceramide-free ghost nanoliposomes (Ghost) for up to 24 h. (**A**) Fixed cells were stained with a mixed lineage kinase domain-like protein (MLKL) antibody (red) and 4’,6-diamidino-2-phenylindole (blue). Imaging was performed by confocal microscopy, and representative images are shown. White arrows show the accumulation of MLKL on the plasma membrane. (**B**–**D**) MDA-MB-231 cells (2 × 10^5^ cells/well) were transfected with 5 nM control or two distinct MLKL siRNAs (MLKL siRNA-1 or MLKL siRNA-2). After transfection for 24 h, cells were treated with 20 µM Ghost or CNLs for 24 h. Cells were harvested and cellular proteins were extracted. Proteins were subjected to Western blotting using antibodies specific for MLKL or β-actin (**B**). The cell death rate was assessed by the trypan blue assay, and data represent the mean ± standard error of three values (**C**). (**D**) MDA-MB-231 cells were transfected with 5 nM control or MLKL siRNAs for 24 h. After transfection, cells were treated with vehicle ethanol or 10 μM C_6_-ceramide for 24 h. Cell death was assessed by trypan blue exclusion assay and data represent the mean ± standard error of three or six values. *, *p* < 0.05; **, *p* < 0.01; ***, *p* < 0.001.

The involvement of MLKL in CNL-induced cell death was assessed in TNBC cell lines. To evaluate the activation of MLKL, its subcellular localization was examined by immunofluorescence microscopy (Figure 5A). MLKL localized to the cytosol under steady-state conditions. In response to CNLs, MLKL relocalized to the plasma membrane in a time-dependent manner. Two siRNA sequences against MLKL were verified to efficiently knockdown MLKL proteins in MDA-MB-231 cells (Figure 5B). In control siRNA-treated cells, CNLs were confirmed to induce cell death. Importantly, the knockdown of MLKL significantly suppressed CNL-induced cell death (Figure 5C), suggesting its involvement in CNL-induced cell death. The necroptotic effects of CNLs were also observed in TNBC-subclassified MDA-MB-468 cells (Supplementary Figure S2). We then investigated the involvement of C_6_-ceramide in necroptotic cell death because CNLs are a nanoliposomal formulation of C_6_-ceramide. Similar to CNL, 10 μM C_6_-ceramide killed MDA-MB-231 cells, and this was significantly suppressed by the knockdown of MLKL proteins (Figure 5D). These results indicate that C_6_-ceramide in CNLs triggered necroptosis in TNBC cells.

## 4. Discussion

The induction and/or manipulation of necroptosis is a promising therapeutic approach. We demonstrated for the first time that the tumor tissues and cell lines of TNBC were characterized by the high expression of MLKL and the use of CNLs as necroptosis-inducing reagents was therapeutically effective in TNBC cells highly expressing MLKL.

Low expression of MLKL is associated with poor prognosis in cervical squamous cell cancer [32], ovarian cancer [33], gastric cancer [34], and pancreatic adenocarcinoma [35]. Those reports are consistent with our findings on breast cancer (Supplementary Figure S1). MLKL might serve as a candidate tumor suppressor and a potential prognostic biomarker in certain types of cancers. Although the clinical significance of the up-regulated expression of MLKL mRNAs in TNBC currently remains unclear, the understanding could play important roles in predicting the effectiveness of MLKL-activating pronecroptotic therapy for TNBC. 

CNLs were therapeutically ineffective against MDA-MB-453 cells, subclassified as a TNBC cell line. MLKL mRNA expression in MDA-MB-453 cells was the lowest among the TNBC cell lines examined in the present study (Supplementary Table S1), which may have resulted in poor susceptibility to CNLs. Moreover, unknown mechanisms also need to be considered for a more detailed understanding of poor susceptibility.

Since Obeid et al. found that C_6_-ceramide is an apoptotic lipid [15], extensive attention has been focused on the development of ceramide-based therapeutics for cancer [18,21,22]. Several types of liposomal formulations of C_6_-ceramide have been developed: a stealth type, such as CNLs [22], and a receptor-targeted type, such as transferrin-conjugated ceramide liposomes [20]. The chemotherapeutic effects of CNLs are well-conserved across cancer types. The mechanisms by which CNLs induce cell death in cancer cells involve apoptosis [24], necrosis [36], and necroptosis [25]. In the present study, we showed that CNLs and C_6_-ceramide were pronecroptotic in TNBC cell lines; however, the underlying molecular mechanisms remain largely unknown. The metabolic fate of short-chain C_6_-ceramide in cells [37] may need to be considered. C_6_-ceramide is subjected to deacylation by ceramidase to form sphingosine. Free sphingosine, in turn, undergoes ceramide synthase (CERS)-dependent acylation [38,39]. Six isoforms of CERS encoded in human genes exhibit distinct substrate preferences for certain chain-length fatty acyl CoAs, thereby generating well-defined ceramide molecular species with specific acyl-chain lengths [39]. Individual ceramide molecular species may be associated with the execution of a distinct type of cell death. Some ceramide molecular species, including C_16_-ceramide, have been implicated in the execution of apoptosis [40,41]; however, the types of ceramide molecular species involved in necroptotic cell death remain unknown. Further studies that clarify the metabolic fate of C_6_-ceramide in CNLs will be instrumental for revealing the biological roles of ceramide molecular species in necroptosis and developing molecular bases for CNL precision medicine. 

Our preclinical analyses of CNLs were based on a cell model and we did not validate the therapeutic effectiveness of CNLs in a TNBC animal model. A previous study demonstrated that treatment with liposomal C_6_-ceramide reduced the size of tumors in mouse xenograft models of the TNBC cell line MDA-MB-231 [42]. The in vivo effectiveness of CNLs has also been validated in various types of cancers, including gastric cancer [24], hepatocellular carcinoma [43], ovarian cancer [25], and leukemia [44]. In future studies, we will examine the pronecroptotic effects of CNLs in a TNBC xenograft model.

In conclusion, TNBC is characterized by the high expression of MLKL and pronecroptotic therapy utilizing CNLs is a potential therapeutic option for TNBC. The present results provide insights into the development of novel precision medicine for TNBC.

## Figures and Tables

**Table 1:** IC_50_ values of ceramide nanoliposomes (CNLs) in breast cancer cell lines. Individual IC_50_ values of eight breast cancer cell lines for CNLs were determined by GraphPad prism.

Immunohistological Subtype	Cell Line	CNL IC_50_ (µM)
TNBC	BT-20	7.1
	HCC1937	7.2
	MDA-MB-231	8.0
	MDA-MB-453	200.7
	MDA-MB-468	17.6
Non-TNBC	MCF-7	55.2
	T47D	32.6
	ZR751	61.0

## Data Availability

Data are contained within the article and Supplementary Materials.

## Supplementary Materials

The following supporting information can be downloaded at https://www.mdpi.com/article/10.3390/cells13050405/s1, Figure S1: Correlation of MLKL mRNA expression with the progression-free survival probability in breast cancer patients; Figure S2: Ceramide nanoliposomes (CNLs)-evoked cell death; Table S1: Expression of Mixed Lineage Kinase Domain-like Protein (MLKL) mRNA in Breast Cancer Cell Lines.
