# Supplementary material for: Pronecroptotic Therapy Using Ceramide Nanoliposomes Is Effective for Triple-Negative Breast Cancer Cells
Source: Cells. 2024 Feb 26;13(5):405. doi: 10.3390/cells13050405 (PMC10931450; doi:10.3390/cells13050405)
Supplement: Supplementary file 1 [file cells-13-00405-s001.zip › cells-2852692-supplementary.pptx]

## Slide 1
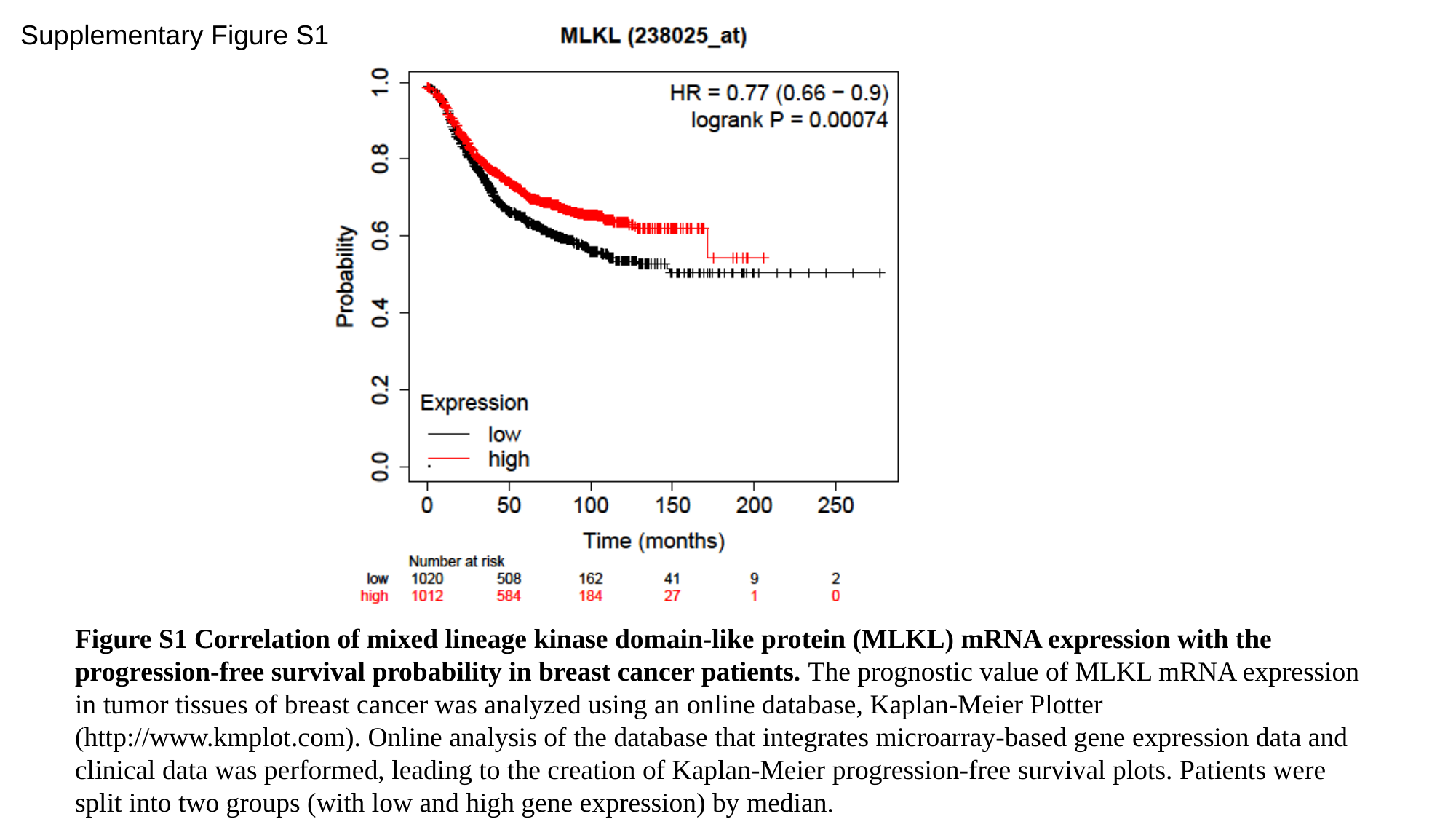

Supplementary Figure S1
Figure S1 Correlation of mixed lineage kinase domain-like protein (MLKL) mRNA expression with the progression-free survival probability in breast cancer patients. The prognostic value of MLKL mRNA expression in tumor tissues of breast cancer was analyzed using an online database, Kaplan-Meier Plotter (http://www.kmplot.com). Online analysis of the database that integrates microarray-based gene expression data and clinical data was performed, leading to the creation of Kaplan-Meier progression-free survival plots. Patients were split into two groups (with low and high gene expression) by median.

## Slide 2
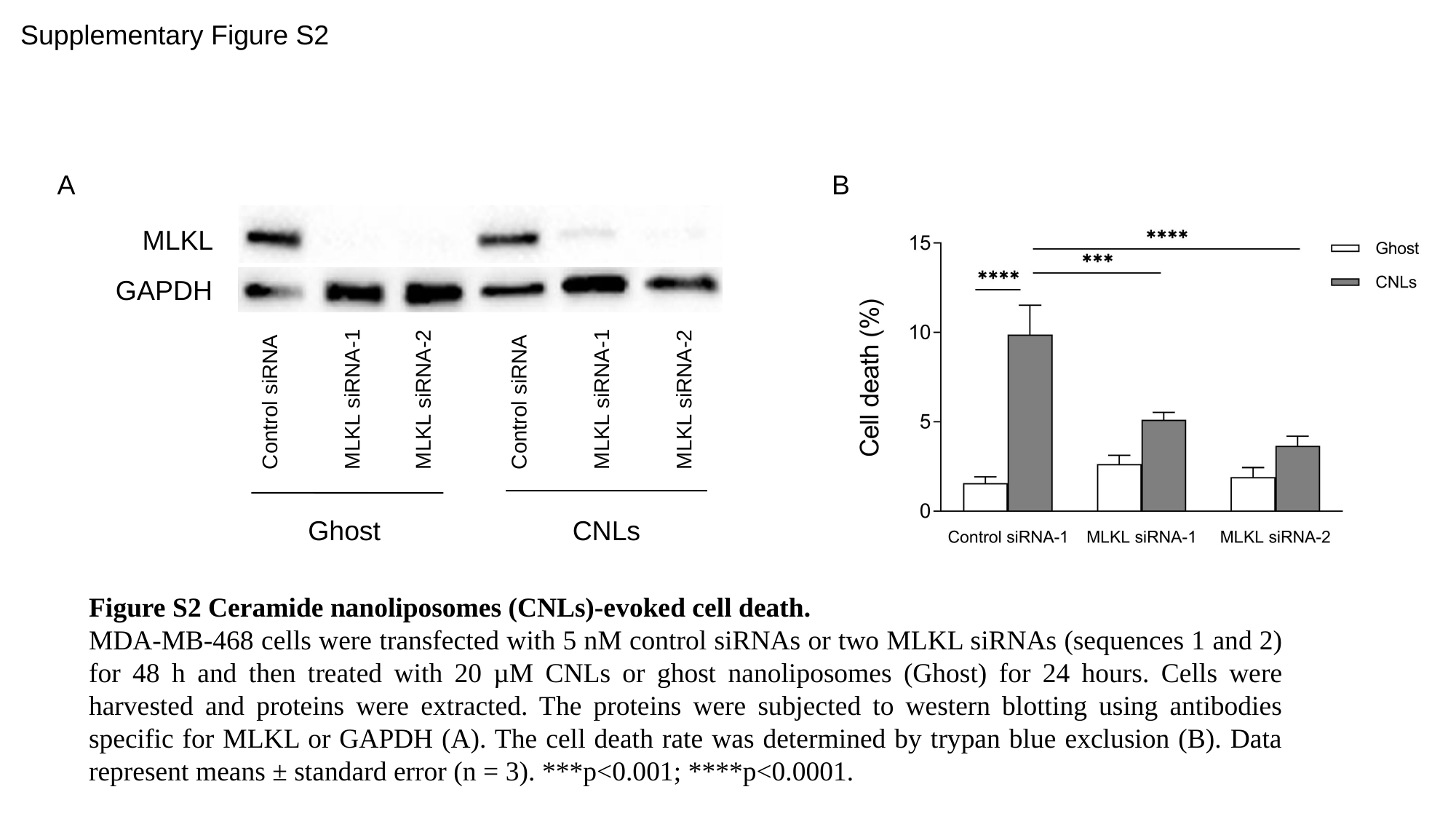

Supplementary Figure S2
A
B
MLKL
GAPDH
MLKL siRNA-2
MLKL siRNA-2
Control siRNA
MLKL siRNA-1
Control siRNA
MLKL siRNA-1
Ghost
CNLs
Figure S2 Ceramide nanoliposomes (CNLs)-evoked cell death.
MDA-MB-468 cells were transfected with 5 nM control siRNAs or two MLKL siRNAs (sequences 1 and 2) for 48 h and then treated with 20 µM CNLs or ghost nanoliposomes (Ghost) for 24 hours. Cells were harvested and proteins were extracted. The proteins were subjected to western blotting using antibodies specific for MLKL or GAPDH (A). The cell death rate was determined by trypan blue exclusion (B). Data represent means ± standard error (n = 3). ***p<0.001; ****p<0.0001.

## Slide 3
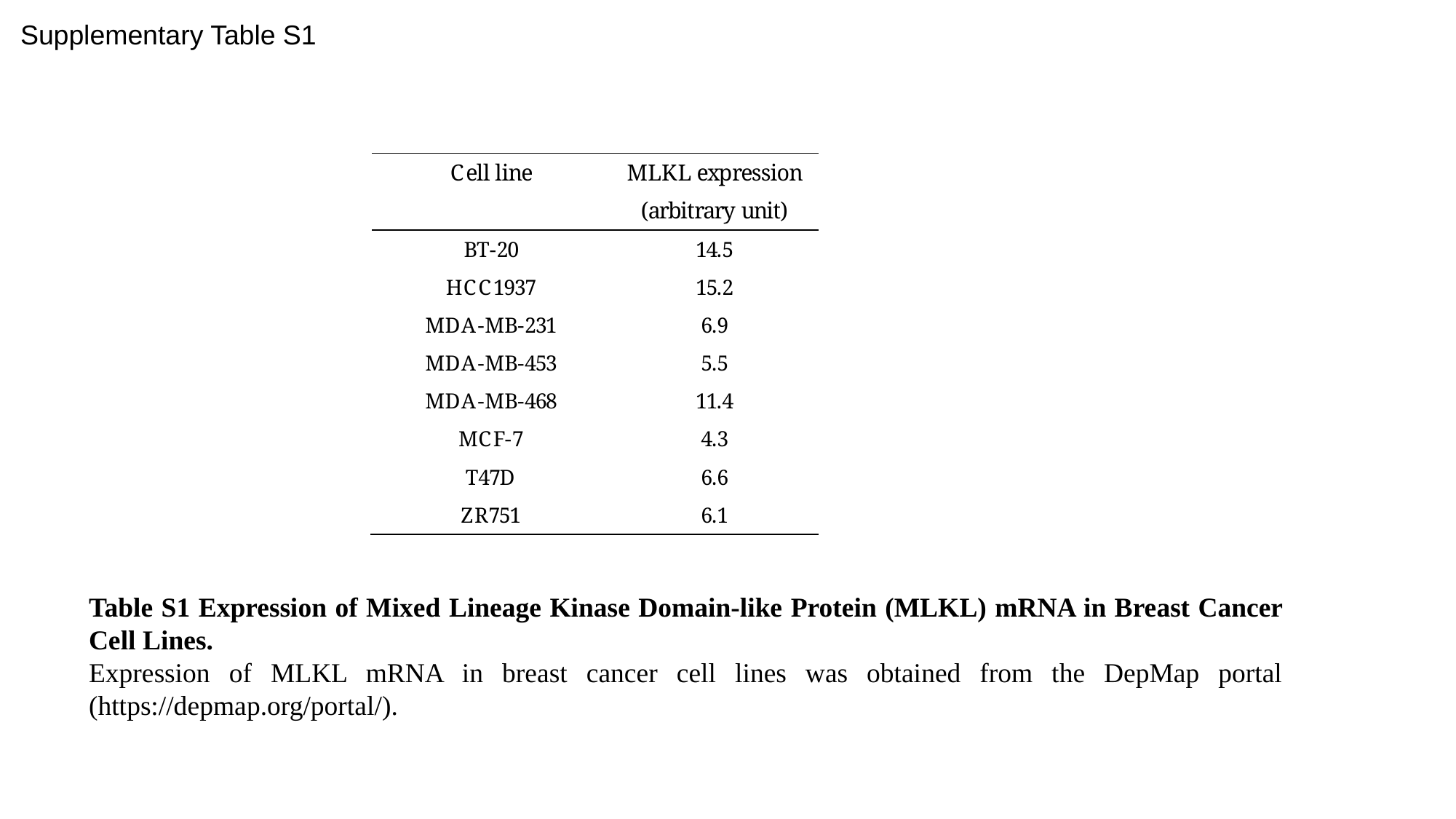

Supplementary Table S1
Table S1 Expression of Mixed Lineage Kinase Domain-like Protein (MLKL) mRNA in Breast Cancer Cell Lines.
Expression of MLKL mRNA in breast cancer cell lines was obtained from the DepMap portal (https://depmap.org/portal/).
